# Appendicitis in Children in a Large Italian COVID-19 Pandemic Area

**DOI:** 10.3389/fped.2020.600320

**Published:** 2020-12-09

**Authors:** Enrico La Pergola, Alberto Sgrò, Federico Rebosio, Daniele Vavassori, Giorgio Fava, Daniela Codrich, Beatrice Montanaro, Ernesto Leva, Jurgen Schleef, M. Cheli, Gloria Pelizzo, Piergiorgio Gamba, Daniele Alberti, Pietro Betalli

**Affiliations:** ^1^Paediatric Surgery Unit, Ospedale dei Bambini V. Buzzi, Milan, Italy; ^2^Paediatric Surgery Unit, Department of Women's and Children's Health, University of Padova, Padova, Italy; ^3^Department of Paediatric Surgery, “Spedali Civili” Children's Hospital, Brescia, Italy; ^4^Department of Paediatric Surgery, Papa Giovanni XXIII, Bergamo, Italy; ^5^Department of Pediatric Surgery, Fondazione IRCCS Ca' Granda Ospedale Maggiore Policlinico, Milan, Italy; ^6^Department of Paediatric Surgery, Institute for Maternal and Child Health, IRCCS Burlo Garofolo, Trieste, Italy

**Keywords:** COVID-19, appendicitis, children, pediatric surgery, incidence of a disease

## Abstract

**Introduction:** The coronavirus disease 2019 (COVID-19) pandemic has dramatically changed the routine activities of pediatric surgical centers, and it determined the reduction of admissions in the pediatric emergency departments (PED). We reviewed the records of patients affected by acute appendicitis (AA) during the COVID-19 pandemic period in a large Italian COVID-19 pandemic area.

**Methods:** Data regarding demographics, age, macroscopic and microscopic findings, and time between symptom onset and PED admission of patients affected by confirmed AA in the period between March and April 2020 were considered. The data were compared with those obtained during the same period of 2019, 2018, and 2017 in the included centers. Data were quoted as median (range) or absolute number. Non-parametric statistical tests were used to compare groups. A *p* ≤ 0.05 was regarded as significant. Since only anonymous data have been used and the data storage meets current data protection regulations, ethical committee approval was not required for this study.

**Results:** Eighty-six patients underwent surgical appendectomy for AA between February 20th, 2020 and April 20th, 2020; 32.5% were complicated appendicitis and 67.5% were uncomplicated. Fifty-three patients were males and 33 were females. Patients' age ranged from 3 to 17 years and the median age was 10 years. The median time between the onset of symptoms and the admission in PED was 1.85 days. The average time between the symptom onset and PED admission was 1.8 days.

**Conclusions:** Although fear from the COVID-19 pandemic determined a delayed diagnosis of serious pediatric diseases, the increasing prevalence and severity of AA were not demonstrated in the most COVID-19-affected areas of Italy.

## Introduction

The epidemic of severe acute respiratory syndrome coronavirus 2 (SARS-CoV-2), causing coronavirus disease 2019 (COVID-19), has rapidly spread worldwide. Italy was the first European country to be affected, with the outbreak estimated to have started in February 2020. At the time of the data collection, Italy was reporting roughly 246,000 COVID-19-positive cases (1) estimating that more than 10% of the Italian population—i.e., ~2 million people—was exposed to the virus (2).

Although the diffusion in all countries of COVID-19 infection was predictable since its beginning in China in November 2019, almost all national health systems were not ready to this dramatic situation. Although this viral infection involves the pediatric population less than adults (3), the pandemic also changed dramatically the management of pediatric patients, and the reduction of pediatric surgical activities in the pandemic period has been already reported (4).

It was our first impression that the admission for abdominal pain in the pediatric population was changing during the pandemic period. In particular, in the initial pandemic period, the authors were empirically observing a decreasing of admissions for acute appendicitis (AA). By contrast, it was a common perception that complicated cases of appendicitis were increasing rather than uncomplicated ones.

The aim of the study was to demonstrate how the pandemic influenced the PED admission and the diagnosis and the evolution of AA in the pediatric population during the pandemic period.

The primary aim of the study was to assess the prevalence of AA in the considered pandemic areas in order to demonstrate if the reduction of cases was real during the pandemic period. The secondary aim was to analyze the number of complicated appendicitis in order to assess if these more serious cases increased during the pandemic period in comparison with the same period of the previous years.

For this reason, the AA data of six referral centers of northern Italy affected by a high incidence of COVID-19 during the pandemic period (20th February 2020–20th April 2020) have been reviewed, and data were compared with those of the same period of the previous 3 years.

## Materials and Methods

Hospital records of patients affected by AA during the COVID-19 pandemic period (since 20th February 2020 until 20th April 2020) and during the same period of 2019, 2018, and 2017 in six north Italian pediatric surgery referral centers of COVID-19 have been reviewed.

The pediatric surgical units involved in the study were Bergamo, Lombardia (Ospedale Papa Giovanni XXIII—center A); Brescia, Lombardia (Spedali Civili—center B); Milan, Lombardia (Buzzi Children Hospital—center C and Policlinico—center D); Padua, Veneto (Azienda Ospedaliero-Universitaria—center E); and Trieste, Friuli-Venezia Giulia (Ospedale Burlo—center F).

Patient demographics, age, macroscopic and microscopic findings, and time between the symptom onset and PED admission were considered. All patients with macroscopic and microscopic non-confirmed appendicitis were excluded. Patients approached with conservative management were not included.

Appendicitis cases were classified as “complicated” (peritonitis, abscesses, perforated appendicitis) and “not complicated.”

We analyzed the days from the symptom onset to PED admission comparing the pandemic period with the same period of the three previous years. Moreover, we compared the days from symptom onset to PED admission in the first month (20th February 2020–20th March 2020) with the second month (21st March 2020–20th April 2020) of the pandemic period.

Data were analyzed including all the series but also considering each center separately. Then, data were divided considering the incidence of COVID-19 in the following areas: Bergamo and Brescia (red zones—>20 per 100,000), Milano and Padua (yellow zones−10–20 per 100,000), and Trieste (white zone— <10 per 100,000) (1).

Qualitative variables were expressed as absolute number (%) and analyzed by Fisher's exact test or Pearson's χ^2^ test. Normality tests with Shapiro–Wilk were conducted for numerical variables, which were expressed as mean (standard deviation, SD) if normally distributed or as median (interquartile range, IQR) and analyzed by one-way ANOVA or Kruskal–Wallis test, respectively. A *p* ≤ 0.05 was considered statistically significant. Data analysis was performed by SPSS Statistics Version 26 (IBM Massachusetts).

## Results

Eighty-six patients underwent surgical appendectomy for AA in the pandemic period considered (20th February 2020–20th April 2020); 121, 96, and 92 patients underwent surgical appendectomy for AA in the same period of the three previous years, respectively (2017, 2018, and 2019). All data regarding the periods investigated are reported in Table 1.

**Table 1:** Demographic and clinical data of patients affected by acute appendicitis between February 20th and April 20th of 2017, 2018, 2019, and 2020.

**Year**	**Area**	**Center**	**Number of appendicitis cases**	**Age**	**Gender (males %)**	**Evaluation after symptom onset (median days)**	**Complicated appendicitis (%)**
2017	Red zone	Center A	33	12.2 (3.0)	23 (69.7)	1.6	8 (24.2)
		Center B	29	9.4 (2.9)	16 (55.2)	1.4	10 (34.5)
	Yellow zone	Center C	24	9.3 (3.5)	14 (58.3)	0.8	5 (20.8)
		Center D	22	9.8 (3.1)	13 (59)	2	7 (31.8)
		Center E	7	10.0 (4.0)	5 (71.4)	2.3	3 (42.8)
	White zone	Center F	6	10.5 (4.4)	5 (83.3)	1.9	2 (33.3)
2018	Red zone	Center A	30	11.1 (3.1)	15 (50)	1.6	10 (33.3)
		Center B	28	9.1 (2.8)	12 (42.8)	1.9	8 (28.6)
	Yellow zone	Center C	17	8.7 (3.3)	7 (41.2)	1	5 (29.4)
		Center D	6	8.2 (4.2)	3 (50)	2.1	3 (50)
		Center E	9	8.2 (3.4)	3 (33.3)	1.9	7 (77.8)
	White zone	Center F	6	9.3 (3.3)	3 (50)	1.8	6 (100)
2019	Red zone	Center A	24	10.6 (3.6)	17 (70.8)	1.5	6 (25)
		Center B	16	10.4 (3.0)	11 (68.7)	1.3	5 (31.2)
	Yellow zone	Center C	22	9 (2.9)	13 (59)	1.7	3 (13.6)
		Center D	15	9 (3.8)	11 (73.3)	1.9	4 (26.7)
		Center E	8	8.6 (3.9)	4 (50)	2.1	4 (50)
	White zone	Center F	7	13.7 (3.6)	3 (42.8)	1.7	1 (14.3)
2020	Red zone	Center A	12	11.9 (2.1)	8 (66.6)	1.7	10 (83.3)
		Center B	18	9.7 (2.8)	9 (50)	1.5	9 (50)
	Yellow zone	Center C	23	9.5 (3.5)	16 (69.5)	1.1	18 (78.3)
		Center D	14	9.6 (4.7)	10 (71.4)	1.8	7 (50)
		Center E	11	8.7 (2.5)	9 (81.8)	1.9	8 (72.7)
	White zone	Center F	8	11.6 (3.8)	3 (37.5)	2	4 (50)

*Data expressed as mean (SD) or median (interquartile range, IQR) or absolute values (%). Different colors for different incidence of Covid infection in the investigated period*.

In the analyzed pandemic period, the surgical technique was a transumbilical laparoscopic-assisted appendectomy (TULAA) in 28 patients, a laparoscopic appendectomy in 51, and an open procedure in other 7 patients. The choice of the surgical procedure depended on the center experience.

Only two patients were affected by COVID-19 in center B, and no cases of infection in other centers were reported. One of the two patients affected by COVID-19 had an uneventful postoperative course. By contrast, the second patient had a complicated appendicitis and required a second procedure for the positioning of abdominal drain (US guided) due to intraperitoneal fluid collection.

Only three patients had no macroscopic or microscopic confirmation of AA and these patients were not considered for the study.

No significant differences between 2020 and the three previous years in terms of prevalence of appendicitis in the large area considered (*p* = 0.21) were found.

Three different areas depending on the incidence of COVID-19 infection have been considered: Bergamo and Brescia (center A and center B) were considered as “red area,” Milan and Padua (center C, center D, and center E) were considered as “yellow area,” and Trieste (center F) was considered alone as “white area.” Considering these different areas, there were no significant differences in terms of prevalence of appendicitis (*p* = 0.2) even if there was a trend of lower prevalence of appendicitis in the red area comparing the same period of the three previous years.

For the secondary aim, the number of complicated appendicitis and those of the same period of the previous 3 years was compared. The statistical analysis did not show any significant differences (*p* = 0.3) even considering “red,” “yellow,” and “white” areas separately (*p* = 0.8).

Finally, the days from the symptom onset to PED admission were analyzed, but no significant differences were found (*p* = 0.4) even between the first and the second months of the pandemic period.

All data and statistical results are reported in Table 2.

**Table 2:** Main results of data analysis per year.

**Year**	**2017**	**2018**	**2019**	**2020**	***p*-value**
Number of appendicitis	121	96	92	86	0.21
Age at onset	10.3 (3.4)	9.5 (3.3)	10.0 (3.5)	10.0 (3.4)	0.44
Evaluation after symptom onset	1.0 (1.0–2.0)	1.5 (1.0–2.0)	1.0 (1.0–2.0)	1.0 (1.0–2.0)	0.95
Complicated appendicitis	35 (28.9%)	35 (36.4%)	23 (25%)	27 (31.3%)	0.38

*Data expressed as mean (SD) or median (IQR) or absolute values (%)*.

## Discussion

AA is the most common abdominal surgical emergency in the pediatric population (5). In the United States, it occurs in approximately 70,000 children per year representing approximately one-third of childhood admissions for abdominal pain (6).

The incidence is age-related from extremely low in the neonatal period to a peak prevalence between ages 12 and 18 years (7). Similarly, the signs and the symptoms in the pediatric population may be different depending on the age of the patient and the intraoperative findings vary widely.

In this paper, we report a large series of AA surgically treated in a wide Italian area with a high incidence of COVID-19 infection in the population. Since its first description, the novel coronavirus disease (COVID-19) rapidly spread all over the world changing dramatically our lives. In the adult population, COVID-19 causes a wide range of disease severity with a fatality rate depending on the countries involved. The spectrum of the disease in children is still debated, but probably, it causes a mild disease with only few cases of patients presenting respiratory distress syndrome or multi-organ failure.

The high incidence of COVID-19 infection in northern Italy caused the cancelation and the delay of routine hospital activities. In particular, many departments, both surgical and medical, were converted to “COVID-19 wards” suspending all planned programs. This happened also for the pediatric surgical departments that had two main problems: first, the increase of the waiting lists (4), and second, the delay in the diagnosis of some acute pathologies.

In this regard, a recent paper from a north Italian pediatric referral center reported that, during Italy's national lockdown for COVID-19, PED admissions showed a substantial decreasing trend in comparison with the same period of the two previous years. Moreover, the same authors reported that delayed PED visits for serious diseases underline the need to prevent delays in PED (8). The considerable reduction in clinic visits may explain this phenomenon.

A case series from Israel reported late diagnosis of seven appendicitis cases resulting from different aspects of the fear from the current global COVID-19 pandemic (9). Similarly, a larger series of adult patients recently reported a significant decrease of AA during the pandemic (10). On the basis of our larger numbers, we can only partially confirm this data. In fact, in our large series, although a trend of reduction of cases of AA has been observed (especially in the very high pandemic diffusion area), the results were not significant.

Snapiri et al. (9) reported a higher incidence of AA complication rates during the same period of the previous year. In our study, we evaluated the differences of incidence of complicated AA between the pandemic period and the three previous years. Although our first impression was of a higher complicated number of cases, the statistical analysis did not confirm our hypothesis.

Of course, several reasons (i.e., the fear of parents to stay in public places, the fear to go to the primary care physician) may justify the delayed diagnosis of AA during the COVID-19 pandemic.

To demonstrate the increasing of delayed diagnosis of AA, we analyzed the time between the symptom onset and the PED admission in the pandemic period, but in this analysis, there were no significant differences in comparison with the three previous years.

Many peripheral centers that also usually treat children with AA sent all patients in our centers during the pandemic period and perhaps this influenced our results. However, this is the first study that analyzed homogeneous data of pediatric patients affected by AA from a wide Italian COVID-19 pandemic area.

In conclusion, COVID-19 is a global pandemic challenging healthcare systems worldwide. The significant decrease in the number of adult patients admitted with AA during the onset of COVID-19 described by Tankel et al. (10) may be justified by the successful medical home treatment of mild appendicitis.

It was also our first impression that PED admission for abdominal pain in the pediatric population was changing during the pandemic period. In particular, in the initial pandemic period, we had the impression that the admissions for acute appendicitis were decreasing.

These thoughts were not confirmed based on our analysis of our large multicentric series of pediatric AA from an area of Italy that has been among the most affected from COVID-19. In particular, there were no significant differences regarding the prevalence and the onset of symptoms of AA in the pediatric population during the peak period of the pandemic.

## Data Availability Statement

The datasets generated for this study can be found in online repositories. The names of the repository/repositories and accession number(s) can be found in the article/supplementary material.

## Ethics Statement

Ethical review and approval was not required for the study on human participants in accordance with the local legislation and institutional requirements. Written informed consent to participate in this study was provided by the participants' legal guardian/next of kin.

## Author Contributions

EL and PB conceived and designed the manuscript. EL and AS drafted and reviewed equally the manuscript. All authors read and approved the final manuscript.

## Conflict of Interest

The authors declare that the research was conducted in the absence of any commercial or financial relationships that could be construed as a potential conflict of interest.

## Abbreviations

PED: pediatric emergency departments
AA: acute appendicitis.
